# Interventional radiology in low- and middle-income countries

**DOI:** 10.1016/j.amsu.2022.103594

**Published:** 2022-04-05

**Authors:** Hashaam Jamil, Waleed Tariq, Muhammad Atif Ameer, Muhammad Sohaib Asghar, Hamid Mahmood, Muhammad Junaid Tahir, Zohaib Yousaf

**Affiliations:** a Lahore General Hospital, Lahore, 54000, Pakistan; b University Hospitals Bristol and Weston NHS Foundation Trust, Weston-Super Mare, UK; c Dow University of Health Sciences–Ojha Campus, Karachi, Pakistan; d Hamad Medical Corporation, Doha, Qatar

**Keywords:** LMIC, Radiology, MRI, Intervention, CT scan

## Abstract

With advancements in imaging techniques, interventional radiology (IR) has found an increased utility in multiple diseases such as ischemic stroke, tissue biopsies, oncology, trauma, etc. The benefit has been twofold in being minimally invasive and improved outcomes. IR in low- and middle-income countries (LMICs) is still in its nascent phase. The many hurdles include poorly structured post-graduate training, cost of procedures, and lack of awareness among referring physicians. There is a significant need to increase the trained specialists' awareness among the medical community and rationalize the cost of procedures in LMICs with careful consideration, planning, and international economic and technical assistance.

Dear Editor

“Interventional Radiology” (IR) refers to a range of techniques that utilize radiological imaging guidance (X-ray fluoroscopy, ultrasound, computed tomography (CT), or magnetic resonance imaging (MRI)) to target therapy precisely. IR treatments comprise minimally invasive techniques. It has replaced several open and laparoscopic surgical procedures. Society of interventional radiology states that with advanced imaging techniques, an interventional radiologist can see and treat different diseases less invasively and with extraordinary precision and accuracy [1]. Nowadays, IR is implemented across multiple medical procedures such as thrombolysis and clot retraction in stroke, tissue biopsies, abscess drainage, central venous access, portosystemic shunting, oncology, infections, uterine fibroids, peripartum complications, vascular diseases, trauma, etc. [2]. IR has improved in outcomes along with reduction in individual and societal costs by shortening in-hospital stay, lowering complication rates, and requirement of general anesthesia [3]. Compared to conventional open surgery, IR causes less pain, less blood loss, lower risk of infection, and faster recovery time [4].

According to the World Health Organization (WHO), more than 4 billion people worldwide lack access to diagnostic medical imaging. This incongruity has widened over time because of a relative lack of progress in low- and middle-income countries (LMICs). There is less than one CT scanner per million population in LMICs compared to almost forty scanners per million population in high-income countries (HICs) [5]. The literature available regarding IR availability in LMICs is scarce, possibly due to the poor IR infrastructure available and insufficient documentation [6]. An intervention radiologist is defined as a board-certified, fellowship-trained physician specializing in minimally invasive, targeted treatments. Interventional radiologists constitute 8.5–11.5% of total radiologists in the US [7].

By comparison, until 2017, there were no formal IR services or a single formally trained interventional radiologist in Tanzania [7]. In 2019, only four hospitals and nine specialists were available across Myanmar to provide IR services [8]. Pakistan has 32 IR fellows who graduated from five different institutes in-country and are practicing in various centers nationally and internationally [9]. India, the second-most populous country with a population of 1.3 billion, has only 596 registered IR specialists depicting that only one IR expert is available per 0.21 million population [10].

LMICs remain unable to benefit from IR in contrast to HICs [2]. The main hurdle in implementing IR in LMICs is nonexistent or poorly structured radiology training. Additionally, IR requires a multidisciplinary approach, a high level of professional expertise, and expensive equipment [11]. The initial setup cost of an IR center and associated ongoing costs make sue of regular IR procedures prohibitive. There is also a lack of awareness among referring physicians due to poor communication with diagnostic radiology and a failure to effectively introduce IR in undergraduate training. Thus, a large proportion of the potential IR patients cannot benefit from it. The procedures in IR are disrupting the previously set norms of treatment and are overtaking procedures from several specialties. Moreover, a lack of proper recognition, suboptimal recruitment pathways, staffing issues, lack of recognized clinical training, and complexity of procedures are some of the challenges in LMICs.

Recently, the novel human coronavirus disease 2019 (COVID-19) pandemic has created significant challenges for health care systems globally. Certain subspecialties like IR entail a greater risk of acquiring and transmitting infection due to close patient contact [12]. The COVID-19 pandemic has also adversely affected IR training due to the cancellation of both urgent and elective procedures resulting in a reduction of training opportunities [13]. The pandemic has resulted in a reduction in diagnostic imaging as a whole, with one study reporting caseload reduction of 16.8–80% with elective activity affected more than urgent work. IR trainees also experienced an 11–51.9% reduction in case volumes [14].

IR training and practice in LMICs is still in its nascent phase, and there is a significant need to promote it at a national and international level. Institutions with accreditation should be open to members of non-accredited institutions to encourage the training. This target can be achieved by increasing the trained specialists and conducting seminars and conferences at multiple levels to increase awareness among the medical community. Active involvement with young physicians and medical students through clinical activities and nonclinical portfolio experience is a key to generating informed and ambitious candidates for the future of IR. The cost of IR procedures should be rationalized, and provision should be provided in public health insurance. With careful planning, healthcare providers in LMICs can be trained to offer lifesaving and minimally invasive procedures. IR is the future of minimally invasive procedures. By training, international collaborative assistance, and tailoring traditional techniques to suit the skill level better and local resources, the implementation of IR in LMICs can be improved. These creative solutions can help in the widespread implementation of IR to LMICs. Finally, with the assistance of international humanitarian organizations and by modifying traditional approaches to basic procedural techniques, radiologists can make significant contributions to promote IR in LMICs.

## Sources of funding

None.

## Consent

Not required.

## Author contribution

H.J, M.J.T, and W.T conceived the idea, M.S.A, M.A.A, and M.J.T retrieved the data, did write up of letter, and finally, H.M, and Z.Y reviewed and provided inputs. All authors approved the final version of the manuscript.

## Registration of research studies

1. Name of the registry: Not required.

2. Unique Identifying number or registration ID: N/A.

3. Hyperlink to your specific registration (must be publicly accessible and will be checked):

## Guarantor

Muhammad Sohaib Asghar and Muhammad Junaid Tahir.

## Ethical approval

Not applicable.

## Funding

None.

## Declaration of competing interest

The authors have no conflict of interest.
